# Unraveling a Major Burden of Orofacial Clefts Analyses: Classification of Cleft Palate Fistulas by Cleft Surgeons

**DOI:** 10.1177/10556656221149521

**Published:** 2023-01-03

**Authors:** Ruben P. Houkes, Johannes A. Smit, N. Lachkar, Raymond Tse, Corstiaan C. Breugem

**Affiliations:** 1 Dept. of Plastic Surgery, Amsterdam UMC, location University of Amsterdam, Emma Children's Hospital, Amsterdam, The Netherlands; 2 Dept. of Plastic Surgery, Seattle Children's Hospital, Seattle, USA

**Keywords:** surgical complications, hard palate, soft palate

## Abstract

**Objective:**

The objective of this study was to investigate how cleft surgeons classify palatal fistulas. We focused on three different anatomical locations (ie, hard palate, soft palate, junction hard/soft palate) to analyze agreement/disagreement at various anatomical locations.

**Design:**

Cross-sectional survey study.

**Participants:**

Participants in an international webinar that focused on palatal fistula treatment were included.

**Intervention:**

Participants were presented with a survey pre- and post-webinar.

**Main outcomes:**

Frequency of used classification systems for classifying oronasal fistulas and the inter-rater reliability of the Pittsburgh classification system.

**Results:**

A total of 141 participants completed the questionnaires prior to the webinar and 109 participants completed the survey after the webinar. In total, four classification systems were used (ie, Pittsburgh, Pakistan Comprehensive Fistula Classification [PCFC], anatomical and ‘other’). The Pittsburgh classification was the most commonly used system in all cases. However, Pittsburgh inter-rater reliability was low (κ = 0.136 pre-webinar, and κ = 0.174 post-webinar). Surprisingly, a substantial shift was observed from the anatomical to Pittsburgh classification after the webinar, indicating increased awareness of the usability of the Pittsburgh classification system.

**Conclusions:**

This study demonstrates a large heterogeneity with regards to the classification of cleft palate fistulas. Interestingly, a shift was observed from the anatomical to Pittsburgh classification after the webinar. However, the inter-rater reliability for using the Pittsburgh classification was low. Classifying palatal fistulas in a homogenous fashion could enhance comparison of primary palate repair and could improve treatment of palatal fistulas.

## Introduction

Cleft palate fistulas can be a complication of cleft palate repair. Three recent reviews indicate an incidence of 4.9–17.9% of the patients after primary palatoplasty, while a higher incidence seems to be correlated to the width of the cleft at the time of primary closure.^1–3^ Fistulas can have a substantial impact on patient quality of life as they may negatively affect speech (eg, nasal air loss on high-pressure consonants, perpetuation of the use of compensatory articulation, cause discomfort when liquids pass through the fistula from the oral to the nasal cavity, and lead to dental and oral hygiene problems, including halitosis.^4–7^ Another problem is the high recurrence risk after secondary surgery.^1^

Palatal fistulas can occur in multiple locations of the palate and in different degrees of severity. They can be located in the anterior section of the palate (ie, located labial-alveolar and in the anterior part of the hard palate), in the mid-palate (ie, dorsal part of the hard palate), at the junction of the hard and soft palate and in the soft palate. Sizes of postoperative fistulas vary widely, from 1-2 millimeters up to multiple centimeters (the equivalent of an unrepaired cleft or larger in the case of prior tissue losses). As Folk et al. mentioned, in order to optimize operative planning, the size, shape and location of the fistula should be described as accurately as possible. The three-dimensional nature of the fistula should be examined through radiological techniques. But also, the nature of the fistula is important information. Is it the result of suboptimal healing after palate repair, or left untreated intentionally? Is it a primary or recurrent fistula? All these aspects are relevant when considering different surgical approaches.^7^

In 2007, a review was conducted by Smith *et al*. to assess different classification systems for palatal fistulas.^8^ They found numerous articles classifying fistulas in different manners and these classification systems were often incomplete or inaccurate. As a result, the authors conceived their own fistula classification system, known as the Pittsburgh classification. It classifies fistulas based on their anatomical location, dividing the palate, alveolus and lip into seven distinct regions. The regions order from zone 1, indicating a bifid uvula to zone 7, indicating a fistula in the labial-alveolar region.

In addition to variations in location of palatal fistulas, each location possibly has numerous surgical approaches and options and specific impacts on speech. In order to compare the different surgical techniques and subsequent outcomes, it is necessary to classify fistulas uniformly. Until consensus is reached, comparison of treatment approaches to palatal fistulas and communication between cleft surgeons and speech pathologists will remain inadequate, hindering progress in decreasing the disease burden of fistulas

The aim of this study was to assess the variety of classification systems used to characterize palatal fistulas. Our second aim was to measure the inter-rater variability of the used classification system. Consequently, we also investigated whether there was a change in the choice of classification system used before and after the special webinar about palatal fistulas.

## Materials and Methods

This cross-sectional study included two surveys that were sent to 242 participants from 74 countries who attended the “Palatal Fistulas: Prevention, Classification & Treatment” webinar hosted in Amsterdam, the Netherlands on July 1 and 2, 2021. The pre- and post-webinar questionnaire consisted of nine patient cases with different fistulas (Supplemental Data Content 1). Participants were asked how they would classify and treat each patient from the presented images. The participants were asked fourteen days prior to the webinar to fill out the first questionnaire. A reminder was sent seven days prior to the webinar and the first survey was closed at the start of the webinar.

After the webinar, participants were again asked to complete a survey with the same questions and images regarding the same nine fistula cases. Reminders were sent after seven and fourteen days respectively. We examined agreement/disagreement of classification between participants pre- and post-webinar. Surveys were included only if a participant answered all the questions. Surveys were created and sent through Castor EDC. Inter-rater reliability was calculated with Fleiss Multirater Kappa scores by using SPSS (Statistical Program for Social Sciences, version 28; SPSS Inc., Chicago, IL, USA). Ethical approval for this study was waived by the local Ethical Committee (W22_158) and participants gave consent for processing all data anonymously for scientific purposes. The reporting was in adherence with the STROBE checklist.

## Results

**Figure 1.**
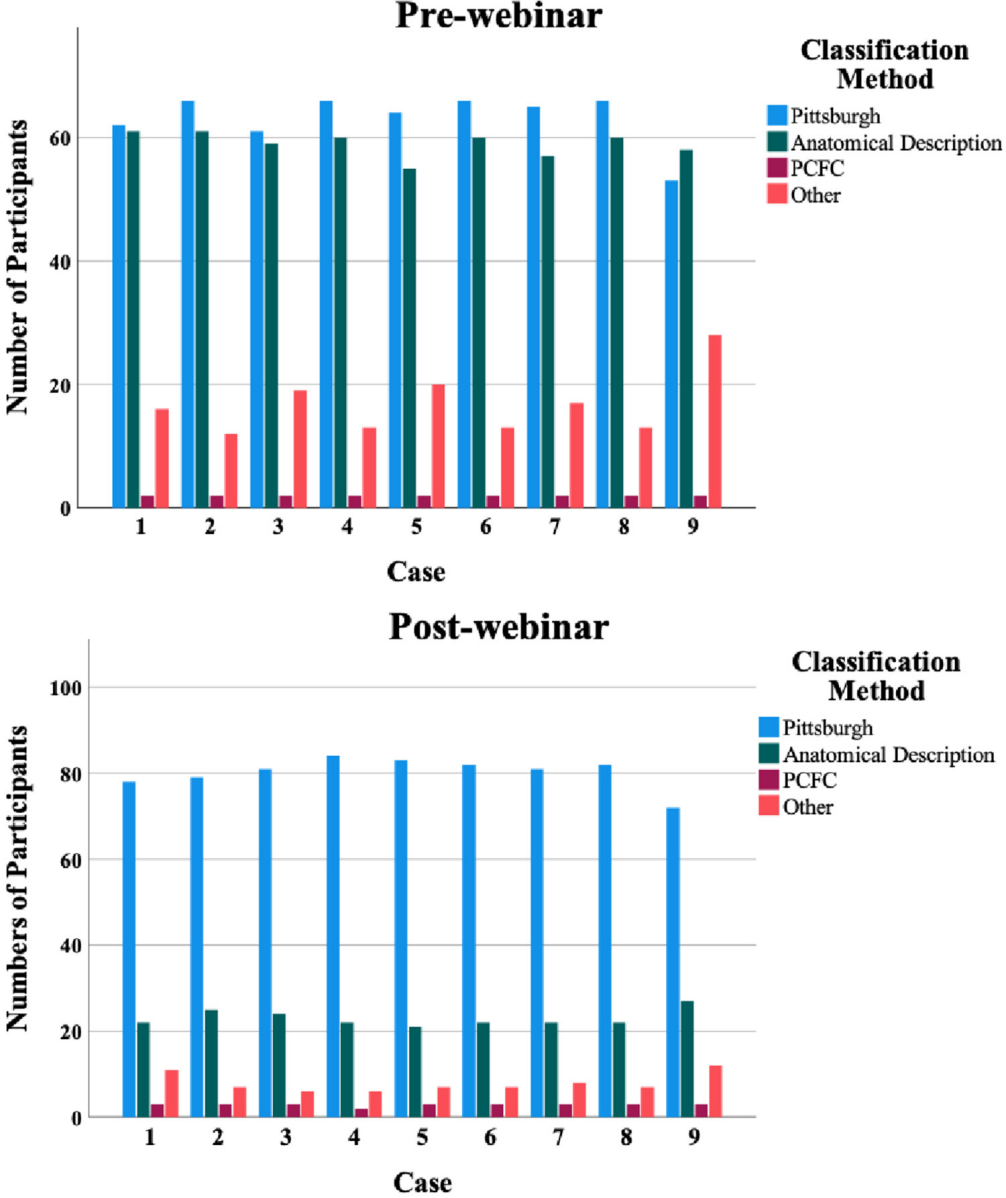
Pre- and post-webinar differences between classification methods used for classifying of fistulas by participants.

A total of 141 participants (response rate 58.3%) completed the questionnaire prior to the webinar, and 114 (response rate 47.1%) responded after the webinar. Pre-webinar, most participants were plastic surgeons (n = 73; 51.8%), followed by maxillofacial surgeons (n = 47; 33.3%) and ENT surgeons (n = 7; 5.0%), pediatric surgeons (n = 5; 3.5%) and other specialties (n = 9;6.4%). Both the Pittsburgh classification system and an anatomical description, i.e., a literal description of what the participant saw in the photograph, were used most often prior to the webinar (Figure 1). On average, 45.4% of the participants classified palatal fistulas according to the Pittsburgh classification system, while 44.8% of participants used an anatomical description to classify palatal fistulas. An average of 1.4% of the participants used the Pakistan Comprehensive Fistula Classification (PCFC) system and 8.4% classified fistulas in another way than previously mentioned prior to the webinar. After the webinar, an average of 71.4% of the participants used the Pittsburgh classification system and an average of 20.4% used anatomical descriptions to classify fistulas. For the PCFC system, an average of 2.7% of the participants indicated that they used this to classify fistulas after the webinar. Another 5.5% of the participants used other methods to classify fistulas. A trend could be identified in which participants classified palatal fistulas more according to the Pittsburgh classification system after the webinar (45.4% versus 71.4%) and tended to use anatomical descriptions and other methods less (respectively 44.8% versus 20.4% and 8.4% versus 5.5%).

Although for each case, a substantial percentage of participants agreed on one Pittsburgh classification, inter-rater reliability was very low (κ = 0.136; 95% confidence interval 0.131–0.142 pre-webinar versus κ = 0.174; 95% confidence interval 0.170–0.179 post-webinar). A more detailed evaluation of the Pittsburgh classification on case level shows great heterogeneity in answers of participants (Supplemental Data Content 2). A similar phenomenon was observed within the anatomical descriptions. Although tendencies by participants towards a certain description (or group within a classification system) were observed, there still exists a large variety among participants regarding the classification of palatal fistulas.

## Discussion

There is great variety in the size and location of palatal fistulas and no standardization in classifying them, even with existing classification systems. Furthermore, due to the relatively low absolute numbers of fistulas per hospital after palatoplasty, it remains a challenge for clinicians to build up experience regarding classifying and treating fistulas. This challenge emphasizes the importance of assessing what classification systems for fistulas are currently being used by clinicians, and determining the inter-rater agreement of these systems.

The participants of this survey most frequently used the Pittsburgh classification system or anatomical descriptions to classify fistulas. A few respondents used the Pakistan Comprehensive classification system.^9^ Compared to the literature, the Pittsburgh classification system is often the primary system used by clinicians.^1,10,11^ Still, anatomical descriptions are also frequently used in scientific literature.^12,13^ The Pakistan Comprehensive Classification System however has not been cited yet to our knowledge in scientific literature besides the manuscript of the original author.^9^

Sitzman *et al*. published a study in 2018 that showed very limited inter-rater agreement for eight surgeons using the Pittsburgh classification system.^14^ This is in line with our findings, where we found κ = 0.136 pre-webinar versus κ = 0.174 post-webinar. Consequently, confusion may occur in communication between different specialists when using the Pittsburgh classification, possibly referring to different types of fistulas, and thereby hampering treatment evaluation.

The impact of fistulas on patient burden is often not discussed and may be undervalued. For example, the Global Burden of Disease project measures, based on global data, the burden of disease of a variety of diseases, including that for clefts.^15^ The burden of clefts is measured through weighing certain aspects of patient disability, namely: 1. “Disfigurement level 1 due to orofacial cleft. Has a slight, visible physical deformity that others notice, which causes some worry and discomfort” (0.011; 95% uncertainty interval, 0.005–0.021). 2. “Disfigurement level 2 due to orofacial cleft. Has a visible physical deformity that causes others to stare and comment. As a result, the person suffering from a cleft is worried and has trouble sleeping and concentrating” (0.067; 95% uncertainty interval, 0.044–0.096). 3. “Disfigurement level 2 and speech problems due to orofacial clefts. (combined disability weights) (0.115; 95% uncertainty interval, 0.076–0.164).”^16^ Although speech problems, which can be caused by fistulas, are taken into the equation, it is noticeable that most emphasis is placed on the physical appearance of clefts. This is understandable, the physical appearance of patients with clefts can have an impact on the patient's mental well-being.^17^ Still, as others have described, the impact of fistulas on patients with clefts can be profound.^4,5^ By not integrating fistulas in the weighing of disability of clefts, measuring the burden of disease will be less accurate.

Some of the limitations of the current study are as follows: First, participants had to classify the patients based on a single photograph. In everyday clinical practice, a better inspection of the fistula from different angles and palpation of the palate is performed for better examination of its location. Due to this limited information, participants were impaired in assessing the location of the fistula. As a result, the degree of consensus between different participants could be lower than in physical outpatient consultation visits, subsequently underestimating the inter-rater reliability of classification systems. Second, although the country of origin from all participants in the webinar were known, we did not include a specific question devoted to the country of origin of respondents. Therefore, we do not know if our respondents’ group is a reliable reflection of the global craniofacial community. Furthermore, the vast majority of respondents were either maxillofacial surgeons or plastic surgeons. No speech therapists did participate in the webinar, and the effect of the size and location of a fistula on speech was not taken into account in this survey. Due to the great impact of fistulas on speech, this was a major limitation of this study. To further increase external validity, all craniofacial team members should be included from countries all over the world.

Although this study primarily focusses on a classification system used for fistulas, we feel that a classification should be created where, after the birth of a child, a cleft classification is created, that could be extended with the possibility to include whether the patient did or did not develop a fistula after primary palatoplasty. In doing so, the impact of a fistula could consider the initial severity of presentation so that we can better understand the burdens of disease and treatment. However, this system should be developed based on a large international consensus, to achieve a higher international implementation rate.

## Conclusion

There is large variation among clinicians concerning the classification of oronasal fistulas, even among those using the same classification system. Consequently, craniofacial specialists do not speak the same language when classifying palatal fistulas or worse, they think they speak the same language but interpret it in different ways. As of yet, no wide implemented classification system for oronasal fistulas with a decent inter-rater reliability exists. To achieve the formation of larger datasets for evaluating the treatment outcomes and assessing the prevalence of different manifestations of fistulas, a more accurate system could be designed, or more training should be provided to physicians involved classifying these palatal fistulas.

## Supplemental Material

sj-docx-1-cpc-10.1177_10556656221149521 - Supplemental material for Unraveling a Major Burden of Orofacial Clefts Analyses: Classification of Cleft Palate Fistulas by Cleft Surgeons Supplemental material, sj-docx-1-cpc-10.1177_10556656221149521 for Unraveling a Major Burden of Orofacial Clefts Analyses: Classification of Cleft Palate Fistulas by Cleft Surgeons by Ruben P. Houkes, Johannes A. Smit, N. Lachkar, Raymond Tse and Corstiaan C. Breugem in The Cleft Palate Craniofacial Journal

sj-docx-2-cpc-10.1177_10556656221149521 - Supplemental material for Unraveling a Major Burden of Orofacial Clefts Analyses: Classification of Cleft Palate Fistulas by Cleft Surgeons Supplemental material, sj-docx-2-cpc-10.1177_10556656221149521 for Unraveling a Major Burden of Orofacial Clefts Analyses: Classification of Cleft Palate Fistulas by Cleft Surgeons by Ruben P. Houkes, Johannes A. Smit, N. Lachkar, Raymond Tse and Corstiaan C. Breugem in The Cleft Palate Craniofacial Journal
