# Supplementary material for: Unraveling a Major Burden of Orofacial Clefts Analyses: Classification of Cleft Palate Fistulas by Cleft Surgeons
Source: Cleft Palate Craniofac J. 2023 Jan 3;61(3):508–12. doi: 10.1177/10556656221149521 (PMC10893769; doi:10.1177/10556656221149521)
Supplement: sj-docx-1-cpc-10.1177_10556656221149521 - Supplemental material for Unraveling a Major Burden of Orofacial Clefts Analyses: Classification of Cleft Palate Fistulas by Cleft Surgeons [file sj-docx-1-cpc-10.1177_10556656221149521.docx]

**Supplementary 1.**

Name and surname:

Age: (groups…..)

Specialty:

Maxillofacial Surgeon

Plastic Surgeon

ENT surgeon

Pediatric Surgeon

Resident surgeon:

Maxillofacial Surgery

Plastic Surgery

ENT surgery

Pediatric Surgery

Other……please mention

How many primary cleft palate surgeries do you perform /year?

- none
- < 10
- 10-25
- 25-50
- > 50 / year

I would always perform a palatal fistula closure in “two” layers

- Yes
- No (one layer only)

What is the most important when you classify palatal fistulas?

1. Should be anatomic
2. Should have functional relationship eg. Impact on speech
3. Should be fast/easy
4. Eligble for reimbursement purposes

Would you indicate what surgery you would do to close the fistula?

**Case 1**


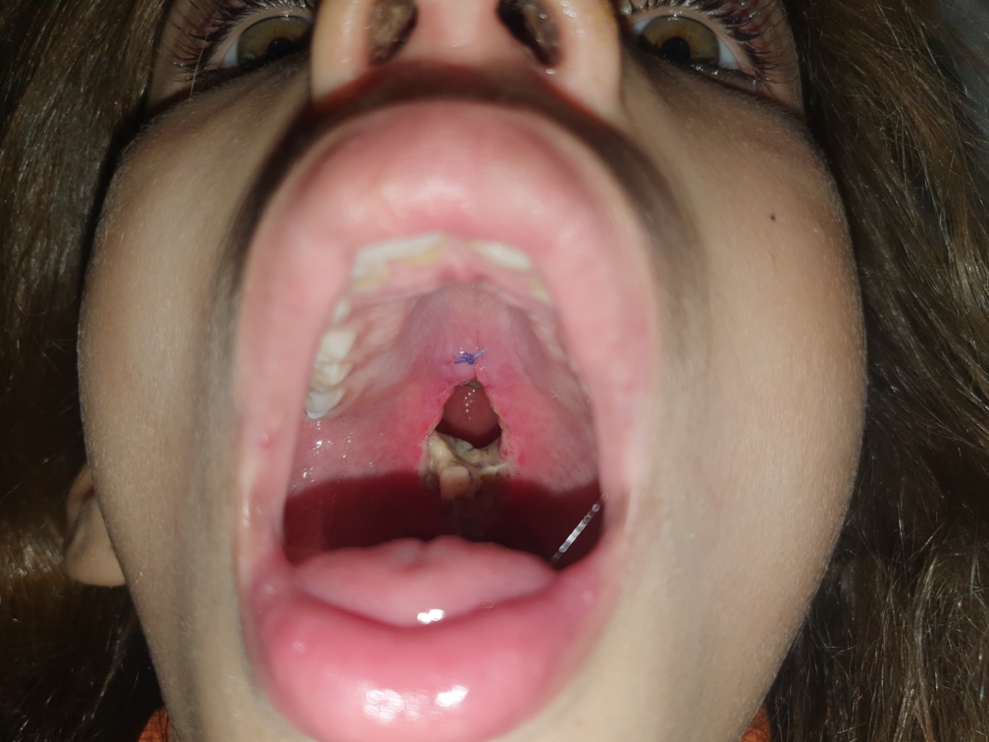


This 4-year-old girl presented late with a cleft palate. Three weeks after palate closure she presents at the outpatient clinic with this fistula.

How would you classify this fistula?

………

**Case 2**

Eight-year-old girl with previously a unilateral cleft lip palate still has this fistula prior to bone grafting of the alveolar cleft. No previous attempts to close fistula in primary palate/ ventral part secondary palate.

Fistula 15 x 12 mm


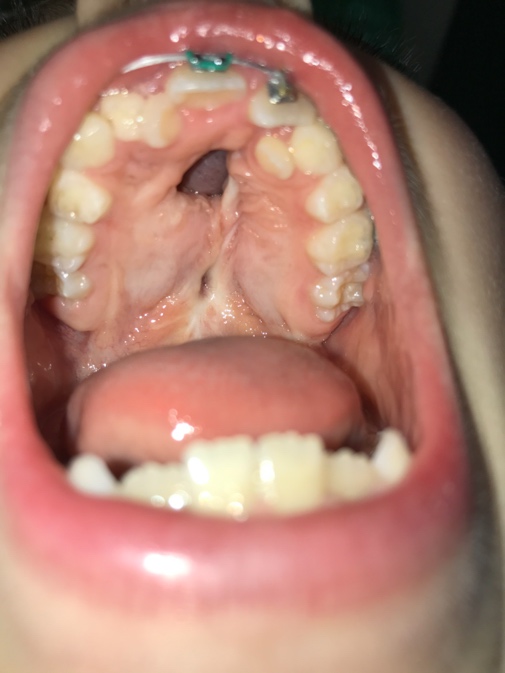


No connection between oral and nasal mucosa

How would you classify this cleft?

……………..

**Case 3**

12-year-old girl with previously a cleft lip palate.

She still has a fistula in the primary palate and half of the hard palate is still open.


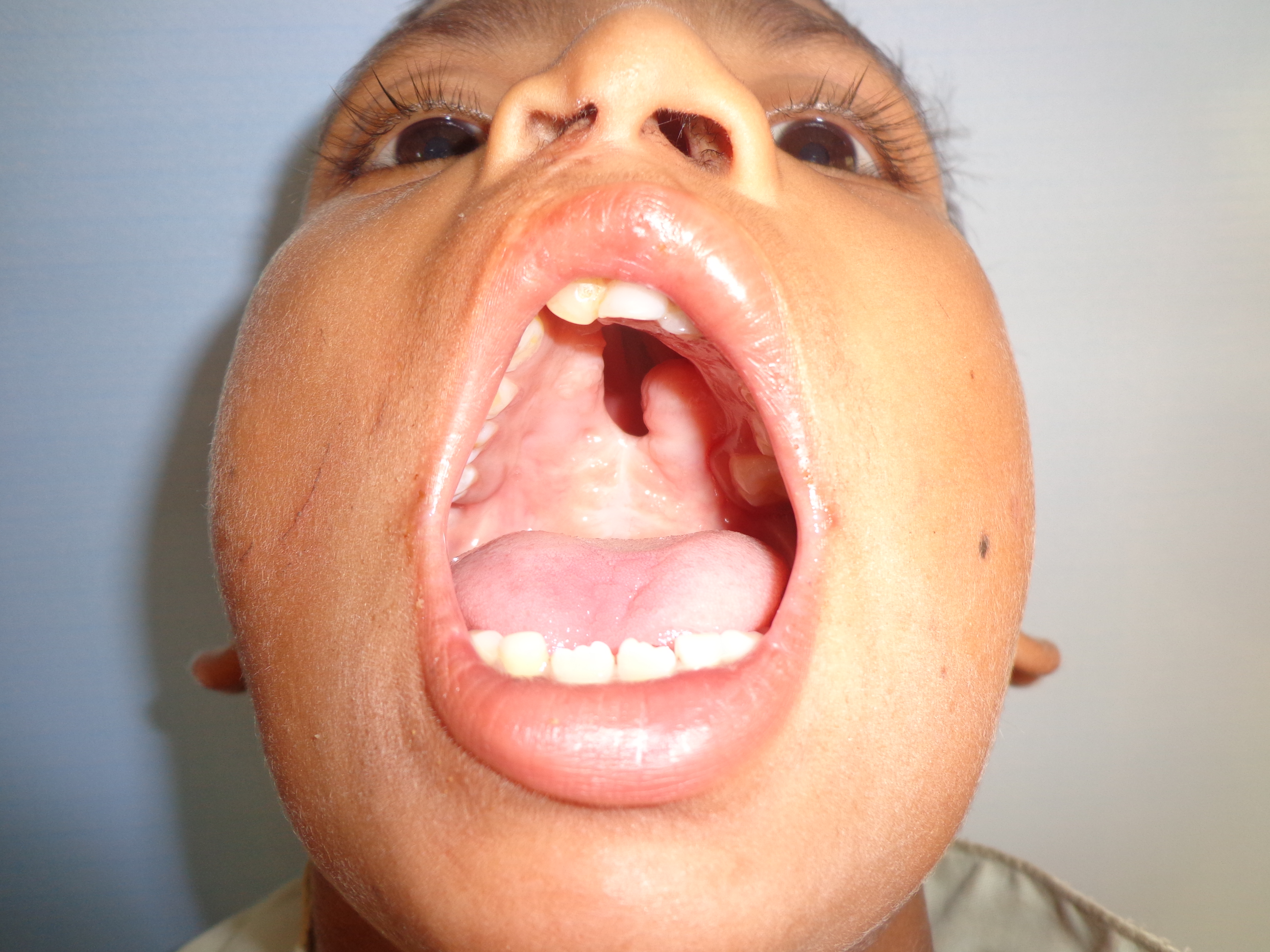


How would you classify this cleft?

……………..

**Case 4**

13-year-old boy with hypernasal speech including a palatal fistula in de middle 1/2 of the hard palate.


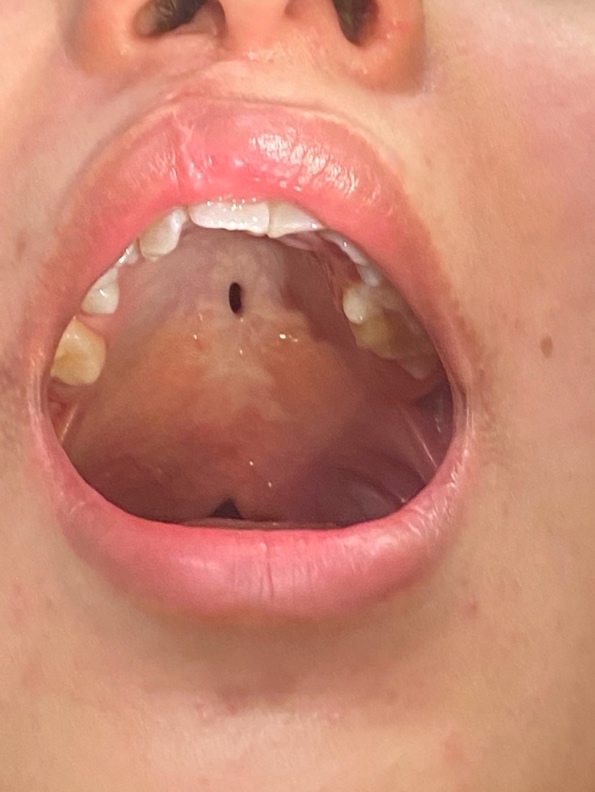


How would you classify this fistula?

……………..

**Case 5**

This 13-year-old boy has a fistula in the ventral 1/3 of the soft palate with mild hypernasal speech.


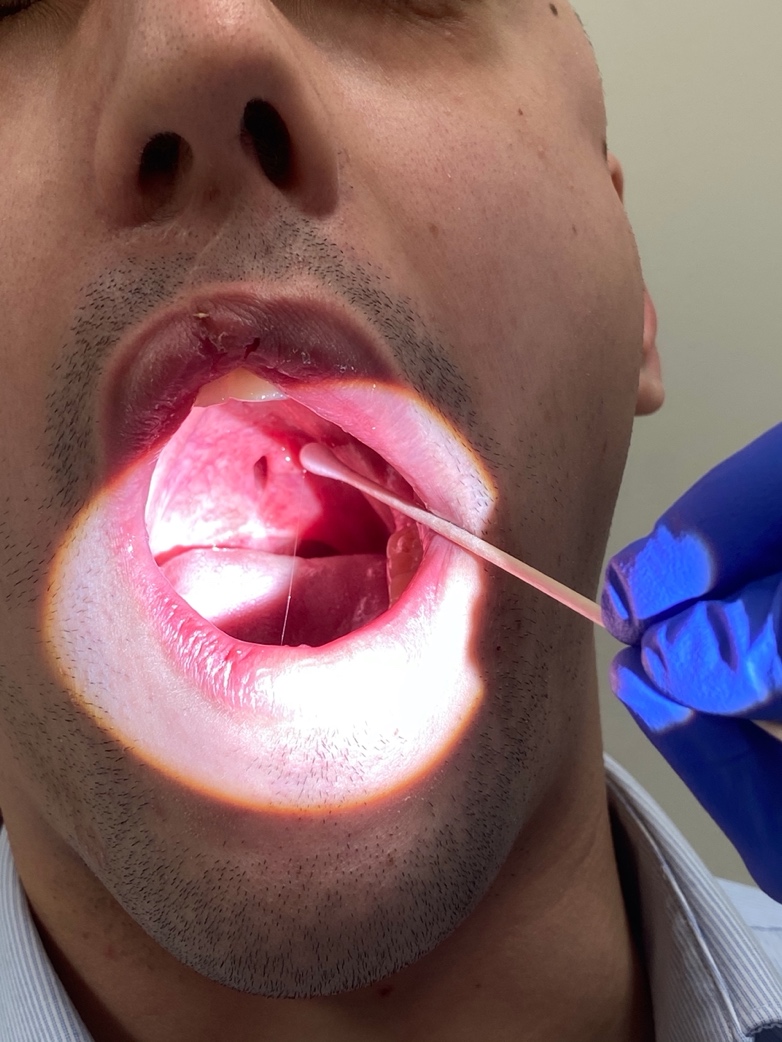


How would you classify this cleft?

……………..

**Case 6**

13-year-old boy with palatal fistula (10 mm) in hard palate and severe VPI


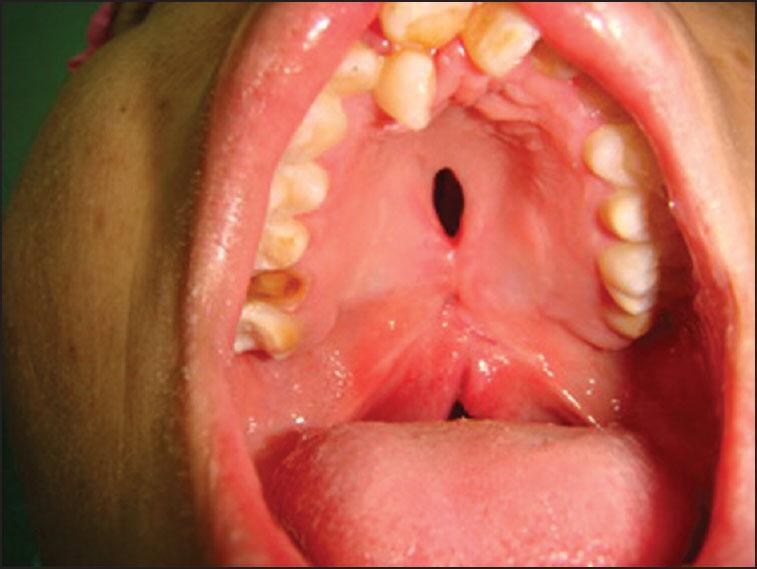


How would you classify this fistula?

……………..

**Case 7**

8-year-old girl with cleft palate in medical history. No vomer flap is available.


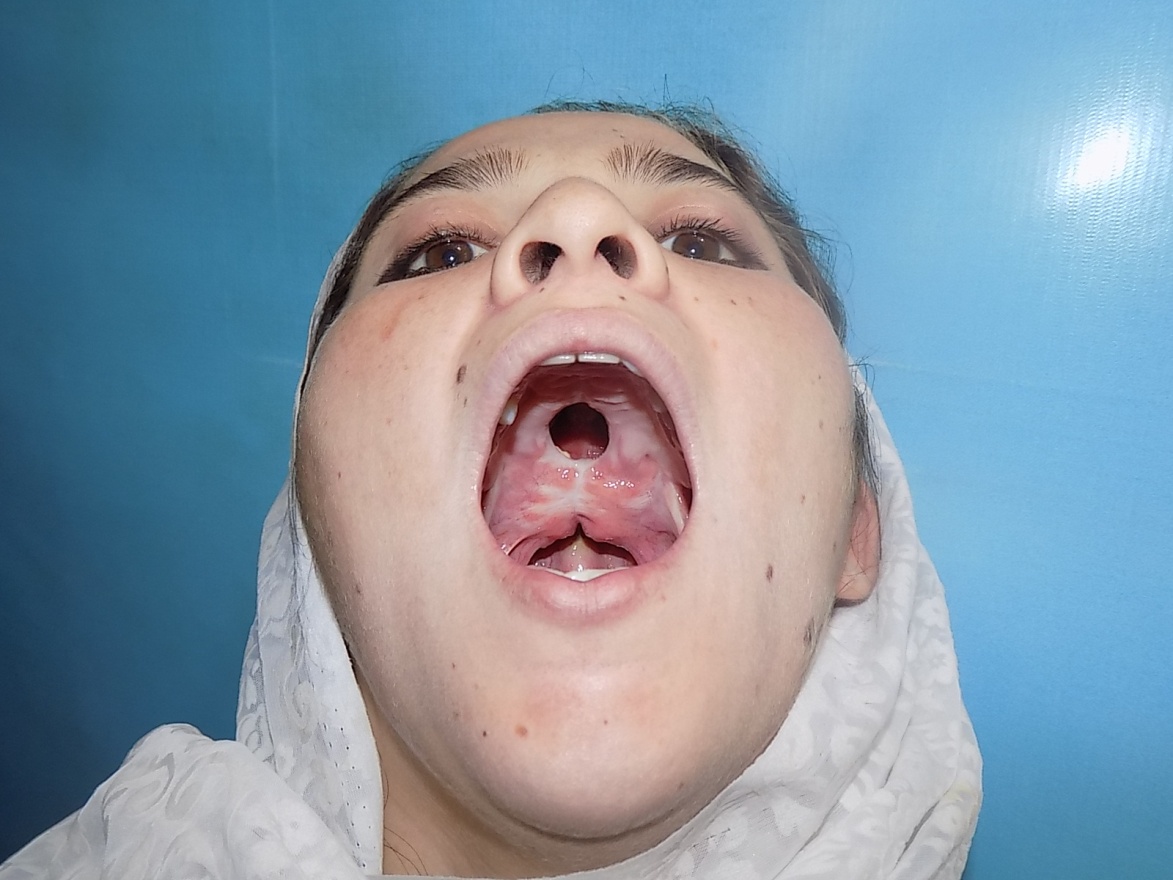


How would you classify this fistula?

…………………….

**Case 8**


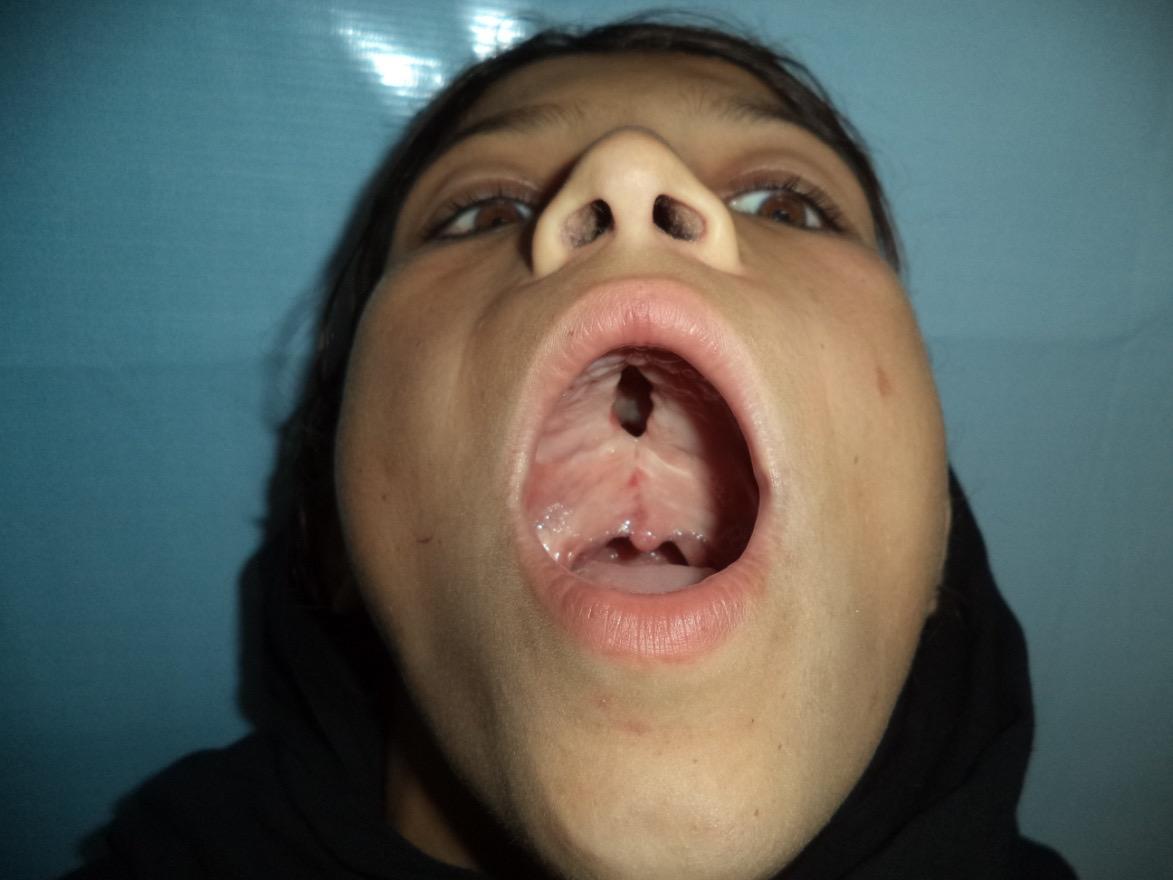


6-year-old girl with a palatal fistula (18 mm in length) in the ventral ½ of the hard palate. The vomer is very small and not possible to use for closure.

How would you classify this fistula?

……………..

**Case 9**

12-year-old girl with large cleft palate. Was referred after primary surgery failed.


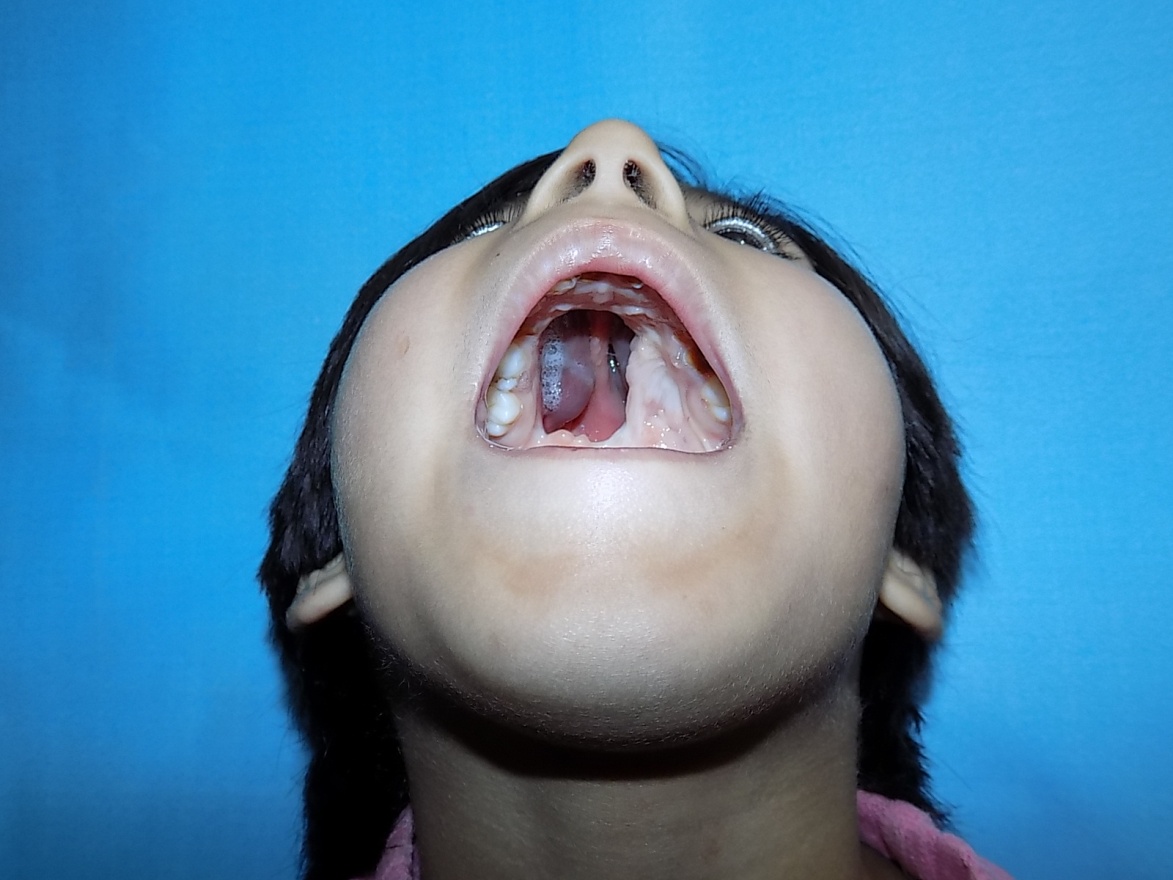


How would you classify this fistula?

……………..
