# Supplementary material for: Unraveling a Major Burden of Orofacial Clefts Analyses: Classification of Cleft Palate Fistulas by Cleft Surgeons
Source: Cleft Palate Craniofac J. 2023 Jan 3;61(3):508–12. doi: 10.1177/10556656221149521 (PMC10893769; doi:10.1177/10556656221149521)
Supplement: sj-docx-2-cpc-10.1177_10556656221149521 - Supplemental material for Unraveling a Major Burden of Orofacial Clefts Analyses: Classification of Cleft Palate Fistulas by Cleft Surgeons [file sj-docx-2-cpc-10.1177_10556656221149521.docx]

**Supplementary 2.**

Classifications of the participants for the nine cases following the Pittsburgh Classification system.

|  | **Pre-webinar** |  | **Post-webinar** |  |
| --- | --- | --- | --- | --- |
| **Case 1** | Pittsburgh 1/2/3  Pittsburgh 2  Pittsburgh 2/3  Pittsburgh 2/3/4  Pittsburgh 2/4  Pittsburgh 3  Pittsburgh 3/4  Pittsburgh 4 | (n=2; 1.4%)  (n=13; 9.2%)  (n=7; 5.0%)  (n=1; 0.7%)  (n=1; 0.7%)  (n=28; 19.9%)  (n=3; 2.1%)  (n=5; 3.5%) | Pittsburgh 1  Pittsburgh 1/2/3  Pittsburgh 1/2/3/4  Pittsburgh 2  Pittsburgh 2/3  Pittsburgh 2/3/4  Pittsburgh 3  Pittsburgh 3/4  Pittsburgh 4  Pittsburgh 4/5 | (n=1; 0.9%)  (n=3; 2.6%)  (n=1; 0.9%)  (n=14; 12.3%)  (n=7; 6.1%)  (n=3; 2.6%)  (n=33; 28.9%)  (n=5; 4.4%)  (n=10; 8.8%)  (n=1; 0.9%) |
| **Case 2** | Pittsburgh 1  Pittsburgh 2  Pittsburgh 2/4  Pittsburgh 3/4  Pittsburgh 3/5  Pittsburgh 4  Pittsburgh 4/5  Pittsburgh 5 | (n=2; 1.4%)  (n=1; 0.7%)  (n=2; 1.4%)  (n=4; 2.8%)  (n=3; 2.1%)  (n=35; 24.8%)  (n=3; 2.1%)  (n=16; 11.3%) | Pittsburgh 2  Pittsburgh 2/3  Pittsburgh 2/4  Pittsburgh 3  Pittsburgh 3/4  Pittsburgh 3/4/5  Pittsburgh 3/5  Pittsburgh 4  Pittsburgh 4/5  Pittsburgh 5 | (n=1; 0.9%)  (n=2; 1.8%)  (n=1; 0.9%)  (n=1; 0.9%)  (n=6; 5.3%)  (n=2; 1.8%)  (n=4; 3.5%)  (n=45; 39.5%)  (n=4; 3.5%)  (n=13; 11.4%) |
| **Case 3** | Pittsburgh 2  Pittsburgh 3  Pittsburgh 3/4/5/6  Pittsburgh 4  Pittsburgh 4/5  Pittsburgh 4/5/6  Pittsburgh 4/5/6/7  Pittsburgh 5  Pittsburgh 5/6  Pittsburgh 6  Pittsburgh 7 | (n=1; 0.7%)  (n=1; 0.7%)  (n=1; 0.7%)  (n=15; 10.6%)  (n=21; 14.9%)  (n=3; 2.1%)  (n=1; 0.7%)  (n=13; 9.2%)  (n=4; 2.8%)  (n=2; 1.4%)  (n=1; 0.7%) | Pittsburgh 2  Pittsburgh 3  Pittsburgh 4  Pittsburgh 4/5  Pittsburgh 5  Pittsburgh 5/6  Pittsburgh 7 | (n=3; 2.6%)  (n=1; 0.9%)  (n=28; 24.6%)  (n=33; 28.9%)  (n=11; 9.6%)  (n=4; 3.5%)  (n=1; 0.9%) |
| **Case 4** | Pittsburgh 1  Pittsburgh 1/3  Pittsburgh 2  Pittsburgh 3  Pittsburgh 3/4  Pittsburgh 4  Pittsburgh 5 | (n=1; 0.7%)  (n=1; 0.7%)  (n=2; 1.4%)  (n=7; 5.0%)  (n=2; 1.4%)  (n=49; 34.8%)  (n=4; 2.8%) | Pittsburgh 1  Pittsburgh 1/4  Pittsburgh 2  Pittsburgh 3  Pittsburgh 4  Pittsburgh 5 | (n=3; 2.6%)  (n=1; 0.9%)  (n=1; 0.9%)  (n=15; 13.2%)  (n=61; 53.5%)  (n=3; 2.6%) |
| **Case 5** | Pittsburgh 1  Pittsburgh 2  Pittsburgh 2/3  Pittsburgh 3  Pittsburgh 3/4  Pittsburgh 4  Pittsburgh 5  Pittsburgh 6 | (n=1; 0.7%)  (n=29; 20.6%)  (n=3; 2.1%)  (n=15; 10.6%)  (n=1; 0.7%)  (n=10; 7.1%)  (n=4; 2.8%)  (n=1; 0.7%) | Pittsburgh 1  Pittsburgh 2  Pittsburgh 2/3  Pittsburgh 3  Pittsburgh 4  Pittsburgh 5 | (n=2; 1.8%)  (n=40; 35.1%)  (n=2; 1.8%)  (n=30; 26.3%)  (n=7; 6.1%)  (n=2; 1.8%) |
| **Case 6** | Pittsburgh 1/2/3  Pittsburgh 1/2/4  Pittsburgh 1/4  Pittsburgh 2  Pittsburgh 3  Pittsburgh 3/4  Pittsburgh 4  Pittsburgh 5 | (n=1; 0.7%)  (n=1; 0.7%)  (n=2; 1.4%)  (n=4; 2.8%)  (n=1; 0.9=7%)  (n=1; 0.7%)  (n=50; 35.5%)  (n=6; 4.3%) | Pittsburgh 1/4  Pittsburgh 2  Pittsburgh 3  Pittsburgh 3/4  Pittsburgh 4  Pittsburgh 5 | (n=2; 1.8%)  (n=2; 1.8%)  (n=5; 4.4%)  (n=1; 0.9%)  (n=71; 62.3%)  (n=1; 0.9%) |
| **Case 7** | Pittsburgh 1/2/4  Pittsburgh 1/3/4  Pittsburgh 1/4  Pittsburgh 2  Pittsburgh 3  Pittsburgh 3/4  Pittsburgh 4  Pittsburgh 4/5  Pittsburgh 5 | (n=1; 0.7%)  (n=1; 0.7%)  (n=1; 0.7%)  (n=1; 0.7%)  (n=8; 5.7%)  (n=10; 7.1%)  (n=41; 29.1%)  (n=1; 0.7%)  (n=1; 0.7%) | Pittsburgh 1/4  Pittsburgh 2  Pittsburgh 2/3/4  Pittsburgh 3  Pittsburgh 3/4  Pittsburgh 4  Pittsburgh 5 | (n=1; 0.9%)  (n=1; 0.9%)  (n=1; 0.9%)  (n=8; 7.0%)  (n=8; 7.0%)  (n=59; 51.8%)  (n=3; 2.6%) |
| **Case 8** | Pittsburgh 1  Pittsburgh 2  Pittsburgh 3  Pittsburgh 4  Pittsburgh 4/5  Pittsburgh 5  Pittsburgh 6 | (n=1; 0.7%)  (n=2; 1.4%)  (n=1; 0.7%)  (n=47; 33.3%)  (n=7; 5.0%)  (n=7; 5.0%)  (n=1; 0.7%) | Pittsburgh 2  Pittsburgh 3  Pittsburgh 3/4  Pittsburgh 4  Pittsburgh 4/5  Pittsburgh 5  Pittsburgh 6 | (n=2; 1.8%)  (n=6; 5.3%)  (n=1; 0.9%)  (n=60; 52.6%)  (n=6; 5.3%)  (n=6; 5.3%)  (n=1; 0.9%) |
| **Case 9** | Pittsburgh 1/2/3/4  Pittsburgh 1/2/3/4/5  Pittsburgh 2  Pittsburgh 2/3/4  Pittsburgh 2/3/4/5  Pittsburgh 3/4  Pittsburgh 3/4/5  Pittsburgh 3/4/5/6  Pittsburgh 3/4/5/6/7  Pittsburgh 4  Pittsburgh 4/5  Pittsburgh 5  Pittsburgh 7 | (n=2; 1.4%)  (n=1; 0.7%)  (n=2; 1.4%)  (n=3; 2.1%)  (n=8; 5.7%)  (n=3; 2.1%)  (n=6; 4.3%)  (n=1; 0.7%)  (n=1; 0.7%)  (n=15; 10.6%)  (n=5; 3.5%)  (n=5; 3.5%)  (n=1; 0.7%) | Pittsburgh 1/2/3/4  Pittsburgh 2  Pittsburgh 2/3/4  Pittsburgh 2/3/4/5  Pittsburgh 3/4  Pittsburgh 3/4/5  Pittsburgh 4  Pittsburgh 4/5  Pittsburgh 4/5/6  Pittsburgh 5  Pittsburgh 6 | (n=1; 0.9%)  (n=1; 0.9%)  (n=13; 11.4%)  (n=6; 5.3%)  (n=8; 7.0%)  (n=7; 6.1%)  (n=22; 19.3%)  (n=8; 7.0%)  (n=1; 0.9%)  (n=4; 3.5%)  (n=1; 0.9%) |
